# Molecular mechanisms and signaling pathways related to brain metastasis in breast cancer

**DOI:** 10.3389/fphar.2025.1585668

**Published:** 2025-03-27

**Authors:** Zhuming Liang, Yanan Mo, Yujiao Zhang, Yanjing Yu, Yinan Ji

**Affiliations:** Department of Breast Surgery, The Affliated Tumor Hospital of Guangxi Medical University, Nanning, Guangxi, China

**Keywords:** breast cancer, brain metastasis, molecular mechanisms, signaling pathways, immune surveillance

## Abstract

Brain metastasis in breast cancer (BCBM) significantly threatens the survival and quality of life of patients, particularly those with triple-negative (TNBC) and HER2-positive subtypes. It involves complex molecular mechanisms and diverse signaling pathways. This review highlights recent research on the molecular mechanisms and signaling pathways of BCBM. The process of BCBM includes several key steps: local infiltration of cancer cells into the bloodstream and subsequent spread to the brain. They must then overcome the blood-brain barrier (BBB) to establish and grow in the brain. Multiple signaling pathways, including PI3K/AKT, STAT3, NF-κB, Notch, and Wnt are involved in this process. Overall, BCBM is a complex disease regulated by multiple molecular mechanisms and signaling pathways. To improve patient survival and quality of life, it is crucial to deepen research into the mechanisms of BCBM and explore new treatment targets and strategies. This will enhance our understanding of BCBM and lead to more effective treatments.

## 1 Introduction

Breast cancer (BC) is the second leading cause of cancer-related deaths in women, after lung cancer (Sung et al., 2021). Brain tumors are a significant threat to human life, classified as primary or metastatic, with brain metastases arising from the spread of malignant tumors from other organs (Aldape et al., 2019). The formation of brain metastases is closely linked to BC, and it is a major cause of death in these patients (Sperduto et al., 2020). Approximately 25% of advanced BC patients develop brain metastases, drastically reducing their quality of life and overall survival (OS) (Darlix et al., 2019). BC is heterogeneous, classified into subtypes based on biomarkers like estrogen receptor (ER), progesterone receptor (PR), human epidermal growth factor receptor 2 (HER2), Ki-67, genomic markers (BRCA1, BRCA2, PIK3CA), and immune markers (tumor-infiltrating lymphocytes, PD-L1) (Allison, 2021). HER2-positive BC and triple-negative breast cancer (TNBC) are more prone to brain metastasis than the luminal subtype (Darlix et al., 2019). The median time from diagnosis to brain metastasis is 28–36 months for HER2-positive BC and TNBC, and 47–54 months for the luminal subtype. Once brain metastasis develops, the median OS is about 1 year (Bailleux et al., 2021). Variations in brain metastasis occurrence among BC subtypes complicate treatment strategy choices.

Breast cancer brain metastasis (BCBM) (Wang et al., 2021) involves several sequential steps. First, cancer cells locally infiltrate breast tissue and enter the circulatory system. Next, they overcome the blood-brain barrier (BBB) to access and colonize the brain. Key signaling pathways such as PI3K/AKT, STAT3, and NF-κB regulate cellular growth, invasion, and metastatic capacity throughout this process. The BBB, comprising endothelial cells, pericytes, the basement membrane, and astrocytes, restricts the entry of most anticancer drugs substances and poses a major obstacle in treating BCBM (Belykh et al., 2020), Understanding these steps and associated signaling cascades is crucial for developing targeted therapies.

## 2 The process of brain metastasis and involved molecular mechanisms

### 2.1 Epithelial-mesenchymal transition (EMT) promotes breast cancer metastasis

Primary BC cells initially initiate metastasis and invasion through epithelial-mesenchymal transition (EMT), a pivotal step in cancer progression (Celia-Terrassa and Kang, 2024). During EMT, cells relinquish epithelial characteristics, including cell-cell adhesion, and adopt a mesenchymal phenotype with elongated morphology and enhanced motility (Dongre and Weinberg, 2019). A key event in EMT is the degradation of the basement membrane by enzymes such as metalloproteinases (MMPs), facilitating the invasion of cancer cells (Liao et al., 2024; Wang and Wang, 2022). EMT is not universal among BC cells; it occurs in a subset, indicating metastatic potential is limited to a select group (Pastushenko and Blanpain, 2019). This heterogeneity suggests that the primary tumor comprises cells at various stages of differentiation, each potentially undergoing EMT at different times (Bakir et al., 2020).

The orchestration of EMT involves numerous molecules and signaling cascades (Liang et al., 2023). Transcription factors, especially the Snail family, play a dominant role by downregulating epithelial markers like E-cadherin and promoting mesenchymal traits (Imodoye et al., 2021). The Twist family also contributes to EMT regulation. ZEB1, another crucial transcription factor, collaborates with Snail and Twist and forms an intricate network governing the EMT process (Mohammadi Ghahhari et al., 2022). Snail and Twist suppress E-cadherin expression, a hallmark of EMT, a function also performed by ZEB1 and modulated by pathways like STAT3 (Sadrkhanloo et al., 2022). These factors are clinically linked to BC prognosis. Snail expression is associated with increased recurrence and metastasis, whereas Twist1 correlates with lower survival rates (Saitoh, 2023). ZEB1 overexpression in TNBC and its interaction with the ER underscore its role in promoting aggressive, invasive phenotypes (Mohammadi Ghahhari et al., 2022).

### 2.2 Entering the circulation

Upon leaving the primary site, tumor cells engage with the extracellular matrix (ECM) and neighboring cells, establishing the tumor microenvironment (TME) (Deng et al., 2024; Zhang X. et al., 2023; Xia Z. et al., 2023; Wang Y. et al., 2022; Li C. et al., 2023). The ECM supports cellular adhesion and migration, housing signaling molecules like cell adhesion proteins and growth factors, facilitating cancer cell migration, invasion, and metastasis (Zhang et al., 2022; Ma et al., 2024) (Wang X. et al., 2022; Soltani et al., 2021). Some cancer cells infiltrate adjacent blood vessels by disrupting the basement membrane and intercellular adhesion, becoming circulating tumor cells (CTCs) (Na et al., 2020). The rise in CTC numbers is a pivotal prognostic indicator linked with distant metastasis survival rates (Pineiro et al., 2020).

Circulating tumor cells (CTCs) face significant challenges in the bloodstream, including immune attacks and hemodynamic stresses, which limit their survival (Na et al., 2020). To evade immune surveillance, CTCs may express PD-L1, which binds to immune cells and inhibits their function (Liu T. et al., 2023; Yilmaz et al., 2023; Wang et al., 2024; Liu et al., 2022). Additionally, CTCs secrete immune-suppressive factors such as TGF-β, impairing immune cell activity (Sanches et al., 2021). They further evade detection by downregulating MHC I molecules, which are essential for presenting tumor antigens to CD8^+^ T cells (Yamamoto et al., 2020; Xie et al., 2024). Hemodynamic forces also play a critical role in CTC migration and survival. High blood flow velocities expose CTCs to elevated shear stress, potentially leading to mechanical destruction, while low velocities enhance interactions with endothelial cells, promoting extravasation (Taftaf et al., 2021). CTCs can form clusters or associate with platelets, creating CTC-platelet aggregates that protect them from shear stress and natural killer (NK) cell-mediated damage (Donato et al., 2020; Chang et al., 2021; Chi et al., 2022). Platelets also provide CTCs with MHC I molecules, shielding them from cytotoxic T cell attacks (Zhou L. et al., 2023; Zhang J. et al., 2023). Despite these survival mechanisms, tumor cells entering circulation face significant obstacles, and only a subset of CTCs successfully establishes distant metastases.

### 2.3 Breaking through the blood-brain barrier

The brain, being highly vascularized, attracts a significant number of circulating tumor cells (CTCs); however, only a small subset successfully crosses the blood-brain barrier (BBB) to form metastases. The blood-brain barrier is primarily composed of endothelial cells, pericytes, the basement membrane, and astrocytes. It plays a critical role in regulating the passage of substances, ensuring central nervous system homeostasis by selectively permitting the transport of essential nutrients while blocking harmful molecules (Terstappen et al., 2021; Hajal et al., 2021). The blood-brain barrier is essential for maintaining brain function and acts as a robust barrier against cancer cell infiltration. The pre-metastatic microenvironment promotes metastasis through mechanisms such as fibronectin deposition, matrix metalloproteinase expression, and interactions with cytokines and extracellular vesicles (Zhang C. et al., 2024; Li et al., 2024; Yang et al., 2024). Within the central nervous system, interactions between CTCs and local cells create a conducive environment for metastasis. After breaching the BBB, tumor cells trigger reactive astrocytes to release plasminogen activators, initiating an anti-tumor response. While these activators eliminate tumor cells in early metastasis, some cells evade destruction by producing anti-plasminogen activators, such as serpins. In advanced stages, reactive astrocytes contribute to a metastatic microenvironment, supporting tumor progression (Ivanova et al., 2023; Wang J. et al., 2023).

The BBB plays a crucial role in limiting cancer cell colonization and metastasis in the brain. Interactions among CTCs, BBB endothelial cells, secreted cytokines, and central nervous system cells are critical determinants of brain metastasis success.

### 2.4 Colonization and growth in the brain

**FIGURE 1:**
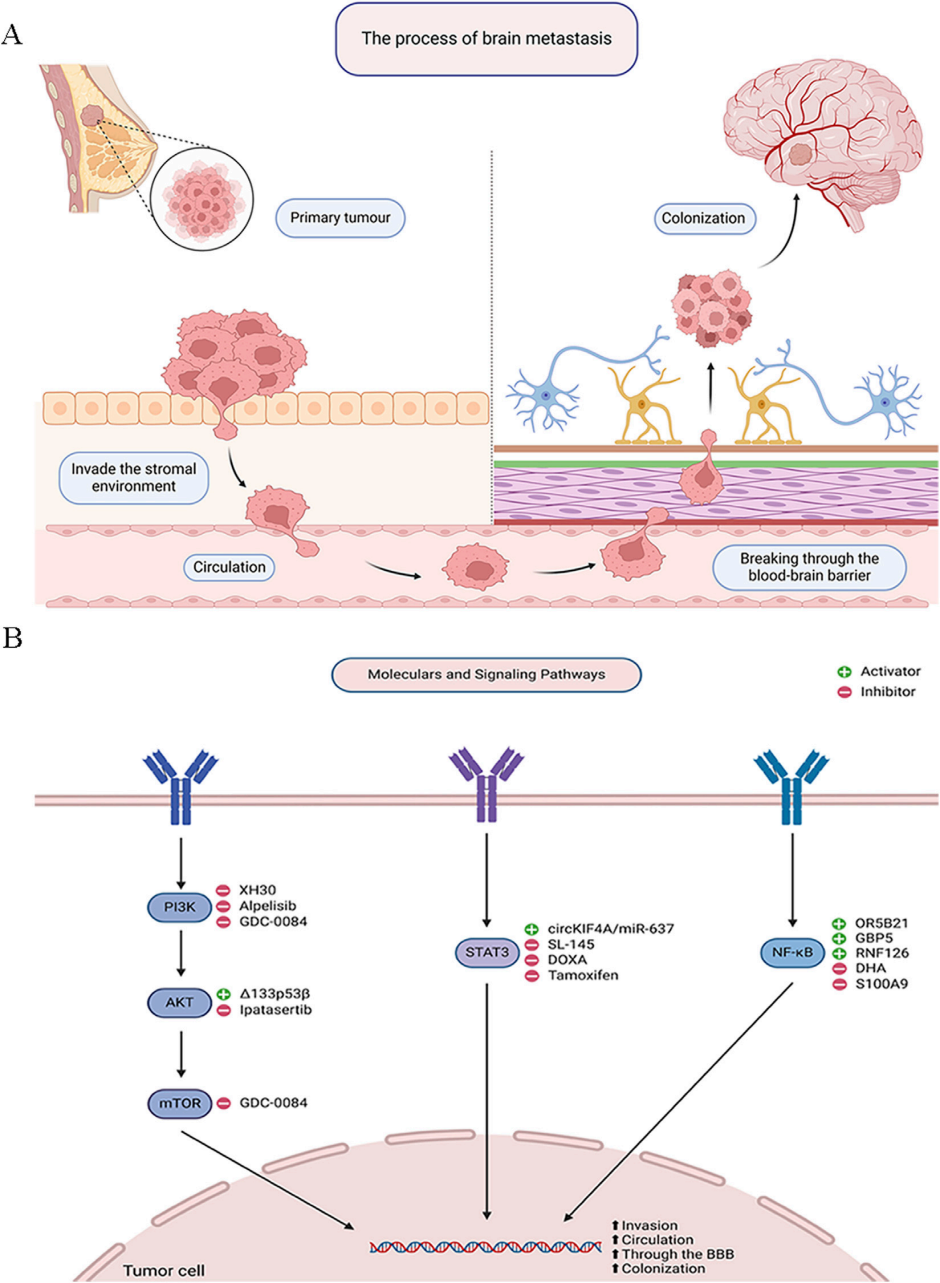
Signaling Pathways in Breast Cancer Brain Metastasis. **(A)** The process of brain metastasis. **(B)** Molecular and signaling pathways.

After breaching the BBB, cancer cells face the challenging task of adapting to the brain microenvironment to establish metastases. Most extravasated cancer cells undergo apoptosis or enter dormancy, with a small fraction resilient to defensive mechanisms, orchestrating vascular remodeling and angiogenesis (Luo et al., 2021). In BC, CTCs activate the HIF-1α signaling cascade to enhance oxygen sensing and cellular adaptation. Additionally, CTCs upregulate anti-apoptotic Bcl-2 family proteins to counter apoptotic signals. However, in brain metastatic lesions of BC, Bcl-2 expression decreases notably compared to primary tumors (Park et al., 2020; Yong et al., 2022). In brain metastasis progression, BC cells activate astrocytes, inducing morphological and functional changes. Activated astrocytes produce chemokines that attract BC cells into the brain parenchyma (Chang et al., 2020). Additionally, astrocytes secrete TGF-β2, which modulates ANGPTL4 expression through SMAD-mediated regulation, facilitating successful colonization (Pedrosa et al., 2018). Growth and angiogenic factors such as VEGF and PDGF promote the formation of new vasculature, providing tumors with ample blood supply (Wang L. et al., 2023). Inflammatory factors are pivotal in diseases progression (Zhai et al., 2024; Xiao et al., 2019; Xiao et al., 2020; Zhang H. et al., 2024; Zhao et al., 2022; Wang et al., 2025). Both CTCs and the brain microenvironment release immune-suppressive factors such as TGF-β and IL-10, inhibiting immune cell activity and impairing their efficacy against tumor cells (Shi et al., 2022). Astrocytes transmit inflammatory cytokines through gap junctions, activating the STAT1 and NF-κB signaling pathways in metastatic cells, promoting proliferation (Zheng et al., 2020). Cancer cells must adapt to the brain’s unique environment to establish brain metastases after crossing the BBB. Astrocytes, growth factors, immune factors, and angiogenesis orchestration play pivotal roles in the evolution of BCBM (Figure 1A).

## 3 Signaling pathways of BCBM

### 3.1 PI3K/AKT signaling pathway

The PI3K/AKT signaling pathway is activated in 43%–70% of BC patients, enhancing BC cell metastatic potential through increased cell proliferation, invasion, and radiation resistance. The PI3K/AKT pathway plays a crucial role in regulating physiological cell functions like proliferation and migration (Zhai et al., 2023). It is also a major altered pathway in many malignancies. Dysregulation of this pathway is associated with treatment resistance, increased angiogenesis, and invasion. AKT, also known as protein kinase B, has three isoforms: AKT1, AKT2, and AKT3. Despite approximately 80% sequence homology, they often have distinct, sometimes opposing physiological roles. For example, AKT1 decreases cell migration and metastasis formation, whereas AKT2 promotes them. AKT1 is mainly responsible for cell proliferation and survival and also has anti-metastatic effects in BC (Miricescu et al., 2020; Glaviano et al., 2023).

Joanna Kempska et al. used *in vitro* cell proliferation and migration assays to evaluate the impact of AKT1 knockout (AKT1_KO) and AKT inhibition with Ipatasertib on MDA-MB-231BR cells. Their findings showed that Ipatasertib increased radiosensitivity and reduced cell proliferation in these cells, whereas AKT1 knockout enhanced cell migration, increased clonogenic survival, and decreased radiosensitivity (Kempska et al., 2023). Similarly, Alexandra N. Boix De Jesus et al. demonstrated that Δ133p53β enhances the invasiveness and migratory capabilities of BC cells by activating the AKT pathway, facilitating their traversal of a simulated BBB and increasing the likelihood of brain metastasis (Jesus et al., 2023). Ming Ji et al. found that the PI3K inhibitor XH30 significantly inhibited the proliferation of various brain cancer cells, reduced phosphorylation levels of key proteins in the PI3K signaling pathway, and induced G1 phase cell cycle arrest, while also suppressing tumor growth in a mouse model of lung cancer brain metastasis. Given the prevalence of lung and BC as primary sources of brain metastases, XH30 shows promise as a potential therapeutic for BCBM (Ji et al., 2022). Recent studies highlight the potential efficacy of PI3K inhibitors in treating BCBM, particularly Alpelisib, which is widely studied for its effects on PIK3CA-mutated BC. In the SOLAR-1 trial, Alpelisib combined with the aromatase inhibitor Fulvestrant demonstrated prolonged progression-free survival compared to Fulvestrant alone, especially in patients with PIK3CA mutations (Zhang and Richmond, 2021). Additionally, GDC-0084, a dual PI3K/mTOR inhibitor with significant brain penetration, has shown potential efficacy in clinical trials for PIK3CA-mutated BCBM (Chen et al., 2022).

### 3.2 STAT3 signaling pathway

STAT3, a crucial member of the signal transducer and activator of transcription family, plays a pivotal role in transcriptional regulation within cells. Activated by various extracellular signals such as cytokines and growth factors (Zhang H. et al., 2023), STAT3 translocates to the nucleus and influences the expression of specific genes involved in cell growth, differentiation, apoptosis, and immune responses. This process begins when cytokines like IL-6 bind to their receptors, activating tyrosine kinases such as the JAK family, which then phosphorylate STAT3. Phosphorylated STAT3 forms dimers, migrates to the nucleus, binds to DNA, and regulates target gene transcription (Xia T. et al., 2023; Chen et al., 2024; Zhou JG. et al., 2023). In BC particularly during brain metastasis, abnormal STAT3 activation is closely associated with increased tumor invasiveness and metastatic potential. STAT3 pathway activation enhances tumor cell survival and anti-apoptotic capabilities, aiding adaptation to the hostile brain microenvironment and promoting metastatic lesion formation (Liu et al., 2024).

Zeller et al. found that STAT3 inhibitors significantly reduce BC cell proliferation and migration, especially in astrocyte-conditioned medium simulating the brain environment. This finding underscores the critical role of the STAT3 pathway in regulating BC behavior during brain metastasis (Zeller et al., 2024). Wu et al. revealed that circKIF4A modulates triple-negative breast cancer (TNBC) progression, particularly brain metastasis, via the miR-637/STAT3 axis. Inhibition of circKIF4A significantly suppressed TNBC cell proliferation, migration, and invasion, while STAT3 overexpression reversed these effects. A TNBC xenograft model further confirmed that circKIF4A promotes brain metastasis through this axis (Wu et al., 2024). Targeting the STAT3 pathway has shown promising anti-tumor and anti-metastatic effects in TNBC. For instance, DOXA treatment inhibited STAT3 activation, reducing cell migration and downregulating MMP-2 and MMP-9, key mediators of tumor invasion (Kim et al., 2023). Additionally, reactive astrocytes contribute to drug resistance and brain metastasis in breast cancer (BC) by activating the IL-6/STAT3 pathway. Tamoxifen, by reducing IL-6 expression and STAT3 activation, may counteract brain metastasis by modulating the brain microenvironment (Xu et al., 2020). These findings indicate STAT3 plays a crucial role in BCBM. Thus, targeting the STAT3 pathway has potential clinical value in preventing or treating BCBM.

### 3.3 NF-κB signaling pathway

The NF-κB pathway plays a critical role in breast cancer (BC), particularly in tumor invasiveness and metastasis. Upon activation, NF-κB translocates to the nucleus, where it initiates the transcription of genes involved in cell adhesion, migration, and invasion, thereby promoting tumor progression (Devanaboyina et al., 2022). In BCBM, NF-κB activation is a key mechanism that facilitates immune evasion, enhances tumor cell survival, and supports brain colonization (Sivamaruthi et al., 2023). Studies have demonstrated that overexpression of OR5B21 in BC cells induces epithelial-mesenchymal transition (EMT) via the NF-κB pathway, enhancing invasive and migratory capabilities, particularly in brain metastasis (Li et al., 2021). Similarly, high expression of GBP5 in triple-negative breast cancer (TNBC) correlates with NF-κB activation, promoting invasion and migration, thereby accelerating brain metastasis (Cheng et al., 2021). Furthermore, FAK-mediated activation of the NF-κB pathway enhances cancer cell interactions with the brain microenvironment, increasing survival and growth in the brain. Systemic inhibition of NF-κB has been shown to reduce brain metastasis development, highlighting its potential as a therapeutic target for BCBM (Lorusso et al., 2022).

The NF-κB pathway also contributes to therapy resistance in BCBM. For instance, RNF126 enhances radiation resistance in BC cells by activating NF-κB signaling, and its inhibition with dihydroartemisinin (DHA) significantly increases tumor sensitivity to radiation therapy (Liu W. et al., 2023). Similarly, S100A9 activates the NF-κB pathway through its receptor RAGE, contributing to radiation resistance. Elevated blood levels of S100A9 may serve as a non-invasive biomarker for assessing radiation therapy response, offering a personalized treatment approach for BCBM (Monteiro et al., 2022). These findings underscore the central role of NF-κB in BCBM and suggest that targeting this pathway could provide a promising therapeutic strategy. Moreover, recent studies highlight the complex interplay among the PI3K/AKT, STAT3, and NF-κB pathways in BCBM. PI3K/AKT, a key regulator of tumor progression (Li Z. et al., 2023), interacts with STAT3 and NF-κB to create a pro-metastatic niche. AKT activation promotes STAT3 phosphorylation via mTORC1, enhancing tumor survival and immune evasion (Mo et al., 2024; Marotta et al., 2011).

### 3.4 Notch and Wnt signaling pathway

In BCBM, Notch signaling is increasingly recognized as a critical regulator of tumor cell migration and colonization in the brain (Nam et al., 2008; Qiao et al., 2022). Studies have shown that Notch pathway activation in BC cells enhances BBB disruption, facilitating cancer cell extravasation into the brain parenchyma (Nam et al., 2008). Astrocytes within the brain microenvironment can secrete Notch ligands, further amplifying these signals in metastatic cells (Xing et al., 2013). As a result, BCBM cells gain a selective advantage, manifesting in more aggressive phenotypes and resistance to conventional treatments. In BCBM, aberrant Notch activation has been linked to increased tumor-initiating cell (TIC) populations and therapeutic resistance (Giuli et al., 2019). Besides, Notch signaling can promote the self-renewal of cancer stem-like cells, facilitating their capacity to seed new metastatic lesions in the brain (Giuli et al., 2019). Preclinical evidence suggests that Notch inhibition can reduce the frequency of brain metastatic lesions and sensitize tumor cells to therapies like radiation and chemotherapy (Mollen et al., 2018).

Previous studies have demonstrated that endothelial Wnt/β-catenin signaling plays an essential role in the formation and maintenance of the BBB (Huang et al., 2024), as well as in brain tumorigenesis (Smid et al., 2008). The active WNT/β-catenin signaling contributes to basal breast tumors metastasizing to brain (Fogarty et al., 2005). Thus, the active WNT/β-catenin signaling by BC cells metastasizing to brain could point to mimicry which, if proven, supports the view that the seed grows better in the soil it resembles (Smid et al., 2008). Besides, in BCBM, abnormal Wnt signaling contributes to cancer stemness, enhancing tumor cell self-renewal and resistance to therapy (Kilmister et al., 2022) (Figure 1B; Table 1).

**TABLE 1:** Key signaling pathways in breast cancer brain metastasis (BCBM).

Pathway	Role in BCBM	Representative evidence	Therapeutic implications
PI3K/AKT	Activated in 43%–70% of BC patients, enhancing proliferation, invasion, and radioresistance; Regulates cell survival and proliferation; AKT isoforms (AKT1, AKT2, AKT3) can have distinct, sometimes opposing, roles	Ipatasertib increases radiosensitivity/reduces proliferation in MDA-MB-231BR; Δ133p53β enhances AKT activation and BBB traversal; PI3K inhibitor XH30 reduces tumor growth in a brain metastasis model	PI3K inhibitors (e.g., Alpelisib, GDC-0084) show efficacy in PIK3CA-mutated BC (SOLAR-1 trial), clinical evaluation; Combination strategies targeting multiple pathways (e.g., STAT3, NF-κB) may overcome resistance
STAT3	Critical for transcriptional regulation of genes involved in growth, survival, and metastasis; Abnormal activation fosters tumor invasiveness and adaptation to the brain microenvironment	STAT3 inhibitors significantly reduce proliferation in astrocyte-conditioned medium; circKIF4A/miR-637/STAT3 axis drives TNBC brain metastasis; SL-145 (HSP90 inhibitor) decreases TNBC invasiveness via STAT3 blockade	Targeting STAT3 (e.g., via IL-6/STAT3 blockade with Tamoxifen) may counteract BCBM; STAT3 inhibition can diminish metastatic lesion formation and improve therapeutic response
NF-κB	Regulates genes involved in EMT, immune evasion, and survival; Contributes to metastatic colonization in the brain	Overexpression of OR5B21 induces EMT through NF-κB; GBP5 enhances NF-κB signaling, promoting TNBC invasion; NF-κB inhibition reduces BCBM development	NF-κB pathway modulation (e.g., via inhibitors) can increase radiosensitivity; S100A9 levels may predict radioresistance and serve as a biomarker; Combined inhibition of NF-κB, PI3K/AKT, and STAT3 may yield synergistic effects against BCBM
Notch	Promotes BC cell migration, BBB disruption, and stemness; Astrocyte-secreted Notch ligands sustain brain metastatic cells	Astrocyte-derived Notch ligands enhance tumor cell survival; Notch activation correlates with elevated tumor-initiating cell populations and therapy resistance	Targeted Notch inhibition may reduce metastatic lesions and improve sensitivity to radiation/chemotherapy
Wnt/β-catenin	Essential for BBB maintenance; aberrant activation in BC fosters metastasis; Reinforces cancer stemness, fueling therapy resistance	Endothelial Wnt/β-catenin signaling is crucial for BBB integrity; Basal BC cells often exhibit active Wnt signaling that promotes brain metastasis	Blocking Wnt signaling could impair metastatic niche formation; Potential combination strategies to target stemness and overcome treatment resistance

## 4 Conclusion

Breast cancer brain metastasis (BCBM) is a severe and life-threatening complication in breast cancer (BC) patients, involving a multi-step process that includes local invasion, circulation, blood-brain barrier (BBB) penetration, and brain colonization. This process is regulated by key signaling pathways such as PI3K/AKT, STAT3, NF-κB, Notch, and Wnt, which influence tumor cell survival, migration, immune evasion, and therapeutic resistance. Understanding the regulatory mechanisms of these pathways in BCBM can elucidate the molecular basis of the disease and provide new therapeutic targets. However, challenges such as the BBB and the complex TME complicate treatment efforts. Current strategies combine systemic and local therapies, with targeted therapies showing promise in some patients. Nevertheless, resistance to treatment remains a significant hurdle, highlighting the need for more effective therapeutic approaches.

Future research on BCBM is expected to focus on multidimensional *in vivo* and *in vitro* studies, including epigenetics, to uncover new treatment insights. Advances in single-cell analysis will enhance our understanding of interactions between cancer cells and the brain microenvironment, while biomarker-based research will facilitate early detection and personalized treatment strategies. Additionally, innovative drug delivery systems and nanotherapies hold promise for overcoming the challenges posed by the BBB and TME. In conclusion, BCBM represents a critical complication in BC patients, and its treatment faces numerous challenges. Ongoing research will deepen our understanding of its mechanisms and lay the groundwork for developing more effective therapies to improve patient outcomes.
